# Periodontal CD14 mRNA expression is downregulated in patients with chronic periodontitis and type 2 diabetes

**DOI:** 10.1186/s12903-015-0118-3

**Published:** 2015-11-18

**Authors:** Dustin C. Hedgpeth, Xiaoming Zhang, Junfei Jin, Renata S. Leite, Joe W. Krayer, Yan Huang

**Affiliations:** 1 Department of Stomatology, James B. Edwards College of Dental Medicine, Medical University of South Carolina, Charleston, SC 29425 USA; 2 Center for Oral Health Research, James B. Edwards College of Dental Medicine, Medical University of South Carolina, Charleston, SC 29425 USA; 3 Division of Endocrinology, Diabetes and Medical Genetics, Department of Medicine, College of Medicine, Medical University of South Carolina, 114 Doughty Street, Charleston, SC 29425 USA; 4 Ralph H. Johnson VA Medical Center, 114 Doughty Street, Charleston, SC 29401 USA; 5 Laboratory of Hepatobiliary and Pancreatic Surgery, Affiliated Hospital of Guilin Medical University, Guilin, 541001 Guangxi People's Republic of China

**Keywords:** Periodontitis, CD14, TLR4, Diabetes, Gene expression

## Abstract

**Background:**

Patients with type 2 diabetes mellitus (T2DM) have increased severity of periodontitis. Toll-like receptor (TLR)4, its co-receptors CD14 and MD-2, and adaptor MyD88 play pivotal roles in lipopolysaccharide (LPS)-triggered tissue inflammation and periodontitis. This study investigated the effects of T2DM and periodontitis on TLR4, CD14, MD-2 and MyD88 mRNA expression in surgically removed periodontal tissues.

**Methods:**

Periodontal tissue specimens were collected from 14 patients without periodontitis and T2DM (Group 1), 15 patients with periodontitis alone (Group 2), and 7 patients with both periodontitis and T2DM (Group 3). The mRNA of TLR4, CD14, MD-2 and MyD88 was quantified using real-time PCR and compared between the groups.

**Results:**

Statistical analysis showed that periodontal expression of CD14 mRNA was significantly reduced across Groups 1, 2 and 3 (*p* = 0.02) whereas the mRNA expression of TLR4, MD-2 and MyD88 was not significantly different among the groups. Furthermore, when patients in Groups 1 and 2 were combined (n = 22), the CD14 mRNA expression was significantly lower than that in patients of Group 1 (*p* = 0.04).

**Conclusions:**

CD14 mRNA expression was downregulated across patients with neither periodontitis nor T2DM, patients with periodontitis alone and patients with both diseases, suggesting that CD14 mRNA expression is associated with a favorable host response or subjected to a negative feedback regulation.

## Background

Gram-negative bacteria are the major pathogens involved in periodontitis, which is characterized by inflammatory tissue destruction of tooth-supporting structures [1]. Studies have established that host immune-inflammatory response to Gram-negative bacteria-derived lipopolysaccharide (LPS) plays a pivotal role in the initiation and progression of periodontitis [2]. As a major breakthrough in periodontal research, Beutler and his coworkers discovered in 1998 that toll-like receptor (TLR)4 is LPS receptor and mediates LPS-triggered signaling transduction [3]. LPS-triggered TLR4 activation leads to an increased secretion of proinflammatory cytokines, mediators and matrix metalloproteinases (MMPs) from a variety of cells including monocytes, macrophages, neutrophils, lymphocytes, and gingival fibroblasts that contribute to periodontal inflammation and tissue destruction [4]. In addition to TLR4, studies have well documented that TLR4 co-receptors CD14 and MD-2 as well as TLR4 adaptor proteins such as myeloid differentiation primary response gene 88 (MyD88) are also vitally involved in LPS-triggered inflammatory signaling [5–8].

It has been well established that periodontitis is more prevalent and severe in patients with poorly controlled type 2 diabetes mellitus (T2DM) than nondiabetic individuals [9–11]. Findings from mechanistic studies indicate that poorly controlled T2DM leads to a hyperinflammatory host response to periodontal microbiota and also impairs resolution of inflammation and repair, leading to accelerated periodontal destruction [10]. In line with these notions, increased periodontal expression of interleukin (IL)-6 and MMP-8 across patients with neither periodontitis nor T2DM, patients with periodontitis alone and patients with both diseases was reported by our group [12–14]. Furthermore, we have also demonstrated through our *in vitro* studies that high glucose enhances LPS-triggered expression of proinflammatory genes in macrophages and gingival fibroblasts [15–19], indicating that T2DM-associated factors such as hyperglycemia enhance periodontal inflammation and tissue destruction and thus exacerbate periodontitis.

In the studies to understand how T2DM increases host responsiveness to LPS, it has been shown that T2DM increases the expression of TLR4 in host cells such as monocytes [20, 21]. Given that TLR4 is the receptor for LPS, it is plausible that upregulation of TLR4 expression on cell surface renders more binding sites for LPS and thus increases cell response to LPS challenge. Therefore, it is important to determine if T2DM increases host responsiveness to LPS by enhancing the expression of TLR4 and TLR4-associated proteins in periodontal tissue. However, the effect of T2DM on periodontal expression of TLR4 and TLR4-associated proteins in patients with periodontitis has not been well established.

In this study, we hypothesized that the expression of TLR4 or TLR4-associated molecules such as CD14, MD-2 and MyD88 are regulated by periodontitis and T2DM. To test our hypothesis, we collected periodontal tissues from patients with or without periodontitis and T2DM at the time of necessary surgeries and determined the mRNA expression of TLR4, CD14, MD-2 and MyD88 using quantitative real-time polymerase chain reaction (PCR).

## Methods

### Patients

Thirty-six patients were selected from a population referred to the Post-doctoral Periodontics Program in the College of Dental Medicine at the Medical University of South Carolina, Charleston, South Carolina. Patients recruited included 14 individuals who did not have periodontitis and T2DM (group 1), 15 patients with periodontitis only (group 2), and 7 patients with both diseases (group 3). The patients in group 1 who required non-periodontal disease-related surgeries, such as crown lengthening and extractions served as controls. All the tissues collected were periodontal tissues taken as collars from the gingival margin to include both epithelium and connective tissue.

The patients from group 2 and group 3 met the following diagnostic criteria for periodontitis [12–14]: A periodontal probing depth (PD) of ≥ 5 mm at two or more teeth, or clinical attachment level (AL) of ≥ 5 mm at two or more teeth. The exclusion criteria were as follows: Patients who were pregnant or unsure about pregnancy status, serum creatinine ≥ 1.6 mg/dL, abnormal hepatic function, hemoglobinopathy (sickle cell trait/hemolytic anemia) interfering with hemoglobin A1c (HbA1c) monitoring, aggressive periodontitis, platelet and coagulation disorders, and/or unwillingness to sign the informed consent form or enter the study. An oral examination including periodontal PD and clinical AL was performed. Six sites (mesio-buccal, buccal, disto-buccal, disto-lingual, lingual and mesio-lingual) were examined for each tooth, excluding third molars. Probing depth was defined as the distance from the free gingival margin to the bottom of the sulcus while gingival recession was defined as the distance from the cementoenamel junction to the free gingival margin. Clinical AL was calculated as the sum of PD and gingival recession. The patients in groups 2 and 3 had periodontal surgery for the treatment of periodontitis and their periodontal tissue, including epithelium and connective tissue, were removed from sites based on the greatest PD and/or AL. The PD and AL reported in this study were related to the surgery sites. The HbA1c test was carried out on all patients to document their glycemic status. T2DM was diagnosed previously for all patients in Group 3. All patients provided informed consent for specimen collection. The study protocol and consent forms were approved by the Medical University of South Carolina’s Institutional Review Board (Approval number: Pro00010000).

### Isolation of RNA and RNA Reverse Transcription (RT)

Total RNA was isolated from periodontal tissue specimens using the RNeasy minikit (Qiagen, Santa Clarita, CA). The first-strand complementary DNA (cDNA) was synthesized with the iScript™ cDNA synthesis kit (Bio-Rad, Hercules, CA) by following the instruction provided by the manufacturer. The reaction was cycled for 5 min at 25 °C, 30 min at 42 °C and 5 min at 85 °C using a PTC-200 DNA Engine (MJ Research, Waltham, MA).

### Quantitative real-time PCR

The RT reaction mixture from the above experiments was then subjected to real-time PCR amplification. The Beacon Designer Software (PREMIER Biosoft International, Palo Alto, CA) was used for designing primers (Table 1). Primers were synthesized by Integrated DNA Technologies, Inc. (Coralville, IA). PCR was carried out in duplicates using a 25 μl reaction mixture that contained 1.5 μl RT reaction mixture, 0.2 μM of both primers and 12.5 μl of iQ™ SYBR Green Supermix (Bio-Rad). The PCR reaction was performed with the iCycler™ Real-Time Detection System (Bio-Rad). Forty cycles consisting of denaturation (95 °C for 10 s) and annealing/extension (56 °C for 45 s) were run. A melt-curve experiment was subsequently performed (55 °C for 1 min and then temperature is increased by 0.5 °C every 10 s) to detect the primer dimers. Data were analyzed with the SmartCycler II software. The average threshold cycle (*Ct*) of fluorescent units was used for analysis. Quantification was calculated using the *Ct* of the target signal relative to that of glyceraldehyde-3-phosphate dehydrogenase (GAPDH) signal in the same RNA sample.

**Table 1:** The primer sequences for real-time PCR

Genes	5’ Primer sequence	3’ Primer sequence
CD14	CCGCTGCCTCTGGAAG	GGCGAGTGTGCTTGGG
TLR4	GTCCTCAGTGTGCTTGTAG	ATCCTGGCTTGAGTAGATAAC
MD-2	CACCATGAATCTTCCAAAGC	CTTGAAGGAGAATGATATTGTTG
MyD88	CGGATGGTGGTGGTTGTCTC	CGCTTCTGATGGGCACCT
GAPDH	CTGAGTACGTCGTGGAGTC	AAATGAGCCCCAGCCTTC

### Statistical analysis

The age, gender and race of the study participants are presented as counts and the continuous variables are presented as medians. The GraphPad Instat 3 software (GraphPad Software, Inc., San Diego, CA) was used for statistical analysis. Nonparametric analyses testing for any differences in continuous variables among groups greater than two were performed using the Kruskal-Wallis procedure. Nonparametric analysis testing for any difference in variables between two groups was performed using the Mann-Whitney procedure. A value of *p* < 0.05 was considered significant. The correlation analysis on the relationship between CD14 mRNA expression and PD, AL or HbA1c was evaluated by Pearson’s method. Our previous studies have indicated that the sample size of 7 or greater provided sufficient power to detect the difference of periodontal mRNA expression of IL-6 and MMP-8 across control individuals who did not have periodontitis and T2DM, patients with periodontitis alone and patients with both periodontitis and diabetes [13, 14].

## Results

### Study population

The clinical data for patient age, gender, race, smoking status, HbA1c, periodontal PD, and AL were presented in Table 2.

**Table 2:** Patients and periodontal disease in three groups

	Group 1: Patients without diabetes and periodontitis	Group 2: Patients with periodontitis alone	Group 3: Patients with both periodontitis and diabetes
Patient number	14	15	7
Age	58 ± 11	56 ± 11	63 ± 6
Gender (male/female)	11/3	10/5	7/0
Race and ethnicity			
Caucasians/African Americans (nonhispanic)	6.0 (12/2)	2.8 (11/4)	0.17(1/6) *
Smoking status (smokers/nonsmokers)	0.4 (4/10) +	1.5 (9/6)	2.5 (5/2)
HbA1c (%)	5.81 ± 0.55	5.60 ± 0.56	8.19 ± 1.24 *
PD (mm)	2.45 ± 0.86 +	4.20 ± 2.00	4.80 ± 2.32
AL (mm)	2.48 ± 0.89 +	5.03 ± 2.48	5.24 ± 2.39

The data are mean ± SD. *p < 0.05 vs group 1 or group 2; +*p* < 0.05 vs group 2 or group 3. *PD* Probing depth, *AL* Attachment level

Age **-** The ages of subjects in groups 1, 2, and 3 ranged from 42 to 79, 40 to 79, and 52 to 71 with a mean ± standard deviation of 58 ± 11, 56 ± 11, and 63 ± 6, respectively. No significant difference between groups was found.

Gender **-** The ratios of male/female gender in groups 1, 2, and 3 were 3.7 (11/3), 2 (10/5), and 7 (7/0), respectively. No significant difference was found between the groups.

Race **-** The ratios of Caucasians vs. African Americans in groups 1, 2, and 3 were 6.0 (12/2), 2.8 (11/4), and and 0.17 (1/6), respectively. Statistical analysis indicated that African Americans in group 3 were significantly more than those in group 1 or group 2, which is consistent with the previous reports that African Americans have a high risk of developing T2DM and periodontitis [22, 23].

Smoking status **-** The smoker/nonsmoker ratios in groups 1, 2, and 3 were 0.4 (4/10), 1.5 (9/6), and 2.5 (5/2), respectively. Statistical analysis indicated that smokers in group 2 and group 3 were significantly more than those in group 1, which is consistent with the previous reports that smoking is associated with a high risk for periodontitis [24].

Periodontitis - The PD in groups 1, 2 and 3 was 2.45 ± 0.86, 4.20 ± 2.00 and 4.80 ± 2.32 mm, respectively. The AL in groups 1, 2 and 3 was 2.48 ± 0.89, 5.03 ± 2.48 and 5.24 ± 2.39 mm, respectively. A significant difference of PD and AL was found between group 1 and group 2 or group 3, but no significant difference of PD and AL was found between group 2 and group 3.

Diabetes - All patients in group 3 reported having T2DM. The mean HbA1c in Groups 3 ranged from 7.0 % to 10.7 % with a mean ± standard deviation of 8.19 ± 1.24 % that is significantly higher than 5.81 ± 0.55 % and 5.60 ± 0.56 %, respectively, in Group 1 and Group 2. Four of the seven patients were prescribed metformin alone or in combination with Glucotrol (Glipizide, Pfizer) or NPH insulin (Humulin, Eli Lily and Company). One patient was prescribed insulin Glargine (Lantus, Sanofi-Aventis), one patient was prescribed insulin Aspart (NovoLog, Novo Nordisk), and one patient did not report taking any medication for diabetes.

### Periodontal expression of CD14, TLR4, MD-2 and MyD88 in three groups

The expression of CD14, TLR4, MD-2, and MyD88 in surgically removed periodontal tissues was successfully quantified using real-time PCR. As shown in Table 3, CD14 expression was highest among four genes in patients without periodontitis and T2DM (Group 1). Our statistical analysis using nonparametric Kruskal-Wallis test showed that the periodontal expression of TLR4, MD-2 and MyD88 had no significant difference among the 3 groups. Interestingly, CD14 expression was found to be significantly downregulated across Groups 1, 2 and 3 (*p* = 0.020). We also compared the CD14 expression between two groups. Results showed that although CD14 expression in Group 2 (patients with periodontitis alone) is lower than that in Group 1 (patients without both periodontitis and T2DM), the difference is not statistically significant. However, when the patients in Group 2 were combined with Group 3 (patients with both periodontitis and T2DM), the CD14 expression was significantly lower than that in Group 1 (*p* = 0.04) (Table 4). Furthermore, we found that CD14 expression in Group 3 is significantly lower than that in Group 1 (*p* = 0.003), indicating that periodontal CD14 expression in patients with both periodontitis and T2DM is significantly lower than that in patients without periodontitis and T2DM.

**Table 3:** Periodontal expression of CD14, TLR4, MD-2 and MyD88 in three groups

	Group 1 (*n* = 14)	Group 2 (*n* = 15)	Group 3 (*n* = 7)	Nonparametric analyses (Kruskal-Wallis test) for any differences in groups	
	Without both periodontitis and diabetes	With periodontitis, without diabetes	With both periodontitis and diabetes	Test statistic	*P*-value
CD14	1.92	1.39	0.68	7.81	0.02
	(0.78, 11.88)	(0.30, 17.80)	(0.17, 1.66)		
TLR4	0.74	0.58	0.74	0.97	0.62
	(0.28, 5.25)	(0.15, 18.31)	(0.14, 1.25)		
MD-2	0.46	0.35	0.50	1.49	0.48
	(0.21, 2.96)	(0.04, 5.76)	(0.19, 1.66)		
MyD88	0.81	0.61	0.71	3.32	0.19
	(0.50, 1.21)	(0.22, 2.19)	(0.45, 1.01)		

Data presented are medians (minimum, maximum)

**Table 4:** The difference of CD14 expression between patients in Group 1 and combined patients in Group 2 and Group 3

	Group 1 (patients without periodontitis)	Combination of Group 2 (patients with periodontitis alone) and Group 3 (patients with both periodontitis and diabetes)	Two-tailed
			*P* value
Patient number	14	22	0.04
Median (minimum, Maximum)	1.92 (0.78, 11.88)	1.22 (0.17, 17.80)	

Data presented are medians (minimum, maximum)

### The relationship between CD14 mRNA expression and PD, AL or HbA1c

The relationship between CD14 mRNA expression and PD, AL or HbA1c was statistically analyzed and no significant correlations were found between CD14 mRNA expression and these clinical parameters (Table 5).

**Table 5:** The relationship between CD14 mRNA expression and PD, AL or HbA1c

		PD	AL	HbA1c
CD14 mRNA expression	n	36	36	36
	r	-0.1271	-0.1862	-0.1967
	*p*	0.4602	0.2770	0.2500

*PD* Probing depth, *AL* Attachment level

## Discussion

Our current study demonstrated that CD14 mRNA level was significantly reduced across patients with neither periodontitis nor T2DM, patients with periodontitis alone, and patients with both periodontitis and T2DM, suggesting that periodontitis and T2DM negatively regulates CD14 gene expression in periodontal tissue. Our findings are consistent with those by Jin et al., who reported that the CD14 protein level in gingival biopsy tissues from patients with periodontitis was significantly lower than that in subjects without periodontitis [25]. They also reported that the level of secreted CD14 in gingival crevicular fluid (GCF) was lower in patients with more deep pockets than that in patients with less deep pockets [26]. Since their studies were focused on CD14 protein level in periodontal tissue, it remains unclear if the decreased CD14 protein level is the result of downregulation of CD14 mRNA expression that may suggest the involvement of transcriptional regulation of CD14 expression. Our current study has provided the new insight into the mechanisms involved in the regulation of CD14 expression.

In the current study, we employed real-time PCR technique to quantify the mRNA expression level of TLR4, CD14, MD-2 and MyD88 in periodontal tissue. It is well known that regulation of cytokine expression at the mRNA level via nuclear factor-kappa B (NFκB)-mediated transcriptional activation plays a crucial role in periodontitis [27]. Therefore, studies investigating gene expression at the mRNA levels are important for understanding the molecular mechanisms involved in the pathogenesis of periodontitis. Furthermore, since real-time PCR is a fully quantitative method and well controlled by normalization to the housekeeping gene, the data are valuable for assessment of gene expression. In addition to real-time PCR quantification of gene expression at the mRNA level, immunohistochemistry and immunofluorescence are also commonly employed in the studies of gene expression at the protein level. While these methods have advantages in the characterization of tissue or cell specific gene expression at the protein level, they also have disadvantage as a semi-quantitative approach [28] that may lead to inter-study variability. For example, by using immunohistochemistry, one study showed that periodontitis decreased TLR4 [29] but another study demonstrated that periodontitis increased TLR4 level in the gingival epithelium [30].

CD14 was characterized as LPS receptor in 1990 [31], almost a decade before the discovery of TLR4. While TLR4 has a transmembrane domain for LPS-triggered signal transduction, CD14 lacks the transmembrane domain and is unable to transmit LPS signaling. CD14 is anchored with glycosylphosphatidylinositol (GPI) on the cell surface and expressed as a 55-kDa glycoprotein [32]. In addition to the membrane-bound form (mCD14), CD14 is also expressed as a soluble form (sCD14) by secretion of CD14 without coupling to the GPI anchor or from shedding or cleavage from mCD14 [33]. The major function of CD14 is to serve as an initial receptor for LPS and facilitate the binding of LPS to TLR4/MD-2 complex [34]. In addition, CD14 also recognizes other pathogen-associated molecular patterns such as lipoteichoic acid [35].

Previous studies have shown that CD14 has dual roles in response to Gram-negative bacteria [36]. For examples, one study showed that neutralization of CD14 prevented LPS-induced uncontrolled proinflammatory response and subsequent host death [37], revealing a proinflammatory effect of CD14. Another study, however, reported that when CD14 was blocked, animals infected with Shigella, a diarrheal pathogen, exhibited a 50-fold increase in bacterial invasion and more severe tissue injury compared with animals without CD14 blocking [38], indicating an anti-inflammatory effect of CD14. These studies suggest that while downregulation of periodontal CD14 expression reduces the stimulatory effect of LPS on proinflammatory signaling, it may also impair the innate immunity against LPS-independent pathogens and thus increases tissue inflammation.

Previous studies have also shown the dual roles of CD14 in T2DM. On one hand, it was reported that CD14 expression was significantly associated with T2DM and correlated with serum concentrations of C-reactive protein [39]; On the other hand, it was reported that injection of recombinant human soluble CD14 to diabetic mice increased insulin action [40] and the mice with CD14-deficiency displayed significant glucose intolerance [41], suggesting a beneficial role of CD14 in insulin signaling and glucose homeostatsis.

The finding that CD14 was downregulated in patients with periodontitis may also reveal a negative feedback mechanism by which periodontal tissue of patients with periodontitis prevent further damage from repeated attacks by bacteria-derived LPS. Interestingly, Nemoto et al. reported that CD14 expression on human gingival fibroblasts (HGFs) was markedly reduced by phorbol 12-myristate 13-acetate (PMA)-activated polymorphonuclear leukocytes (PMNs) in a coculture system of HGF and PMNs [42]. Their further investigation showed that as a result of CD14 reduction, LPS-induced production of inflammatory cytokines such as IL-8 by HGFs was suppressed. These findings suggested that while activated PMNs play a crucial role in tissue inflammation, HGFs have a potential negative feedback mechanism to control inflammation by downregulation of CD14 expression.

In addition to CD14, our current study also examined the periodontal expression of TLR4, MD-2 and MyD88. Results showed that the periodontal mRNA expressions of TLR4, MD-2 and MyD88 had no significant difference across the 3 groups. While our current study is the first one focusing on TLR4 mRNA expression, Promsudthi et al. and Rojo-Botello et al. have reported the effect of T2DM and chronic periodontitis on TLR4 protein expression [29, 30]. Promsudthi et al. showed that patients with periodontitis had reduced TLR4 protein in gingival epithelium, but patients with both periodontitis and diabetes had statistically significant higher percentages of TLR4-positive cells as compared with periodontally healthy subjects [29]. Rojo-Botello et al. demonstrated that patients with periodontitis and diabetes had a higher TLR4 protein in gingival epithelium than patients with periodontitis alone. Obviously, these studies focused on the periodontal TLR4 protein expression, but our study aimed at the TLR4 mRNA expression.

It has been well documented that CD14 expression is upregulated by LPS and inflammatory cytokines *in vitro* [43–45]. However, the mechanisms involved in the downregulation of CD14 expression have not been well established. In addition to the inhibition of CD14 expression by PMNs as described above, Imai et al. have reported that transforming growth factor beta (TGFβ)1 is capable of inhibiting LPS-stimulated CD14 expression in macrophages by reducing transcription factor activating protein (AP)-1 [46]. Interestingly, our recent study has shown that periodontal TGFβ1 expression is increased in patients with periodontitis [47]. Therefore, it is likely that TGFβ1 plays a role in the downregulation of periodontal CD14 expression in patients with periodontitis. Nevertheless, it remains unclear how CD14 expression is further downregulated by T2DM and more studies are necessary to elucidate the mechanisms by which periodontal CD14 expression is downregulated by periodontitis and T2DM.

## Conclusions

CD14 expression was downregulated across patients with neither periodontitis nor T2DM, patients with periodontitis alone, and patients with both periodontitis and T2DM. This finding indicates that CD14 expression is associated with a favorable host response or subjected to a negative feedback regulation.

## Abbreviations

TLR: Toll like receptor
LPS: Lipopolysaccharide
MyD88: Myeloid differentiation primary response gene 88, MD-2, Myeloid differentiation factor 2
PD: Probing depth
AL: Attachment level
